# Optimization of a hyperspectral imaging system for rapid detection of microplastics down to 100 µm

**DOI:** 10.1016/j.mex.2020.101175

**Published:** 2020-12-08

**Authors:** Chunmao Zhu, Yugo Kanaya, Masashi Tsuchiya, Ryota Nakajima, Hidetaka Nomaki, Tomo Kitahashi, Katsunori Fujikura

**Affiliations:** a Earth Surface System Research Center, Research Institute for Global Change, Japan Agency for Marine-Earth Science and Technology (JAMSTEC), Yokohama 2360001, Japan; b Marine Biodiversity and Environmental Assessment Research Center, Research Institute for Global Change, Japan Agency for Marine-Earth Science and Technology (JAMSTEC), Yokosuka 2370061, Japan; c Institute for Extra-cutting-edge Science and Technology Avant-garde Research, Japan Agency for Marine-Earth Science and Technology (JAMSTEC), Yokosuka 2370061, Japan

**Keywords:** Plastic pollution, Near-infrared spectroscopy, Macro-photography

## Abstract

Plastic pollution has become one of the most emergent issues threating aquatic and terrestrial ecosystems. However, it is still challenging to rapidly detect small microplastics. Here, we present a method to rapidly detect microplastics using hyperspectral imaging in which we optimized a commercially available hyperspectral imaging system (Pika NIR-640, Resonon Inc., USA). The optimizations included: (1) changing the four-lamp assembly to a symmetrical set of converged-light near-infrared lamps that are placed sideways instead of above the sample stage; (2) adopting a macro-photography technique by applying an extension tube between the camera and the lens, and moving the lens of the hyperspectral camera to the imaging target (working distance of ~3 cm); (3) adjusting the exposure and aspect ratio by tuning the frame rate and scan speed of the imaging system. After optimization, the detection resolution of each pixel improved from 250 µm to 14.8 µm. With the optimized system, microplastics down to 100 µm in size were rapidly detected. This result is promising for the application of our new method in the accelerated detection of microplastics and will contribute to a better understanding of the microplastic pollution situation.

## Graphical abstract

**Figure:**
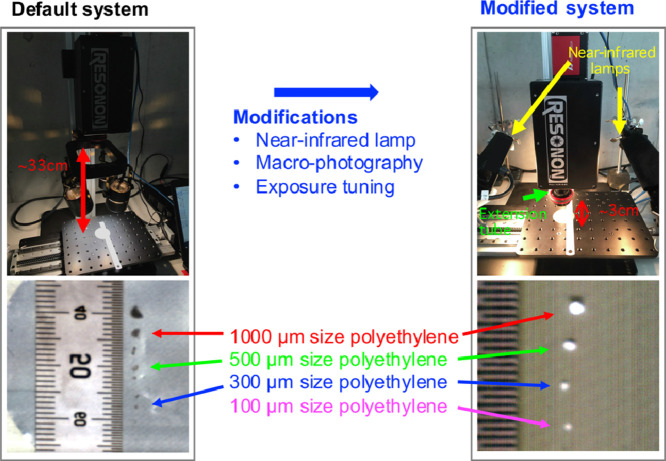

Specifications table

**Table:** 

Subject Area:	Environmental Science
More specific subject area:	Rapid detection of environmental microplastics
Method name:	A rapid microplastic detection system based on hyperspectral imaging
Name and reference of original method:	Hyperspectral Imaging System Resonon Inc., Spectronon Pro Manual (Release 5.3), 2019, http://docs.resonon.com/spectronon/pika_manual/SpectrononProManual.pdf (accessed 18 March 2020)
Resource availability:	Imaging system: Benchtop hyperspectral imaging system (Pika NIR-640 camera equipped), Resonon Inc., USA Software: Spectronon Pro, Resonon Inc., USA Optimization lamp: LN-200CIR, CCS Inc., Japan Extension tube: CMV10, Thorlabs Inc., USA

**Method details**

**Background**

Investigations on microplastics in the environmental samples have been mainly based on sample collection, separation, and quantification in the laboratory [4]. Visual observation, Fourier transform infrared and Raman spectroscopy, and pyrolysis-gas chromatography are major ways of identification. However, these methods are time- and labor- consuming [16]. In comparison, hyperspectral imaging techniques provide a solution for the rapid and convenient quantification of environmental microplastic particles. In this study, we present technical details for the detection of small microplastics using hyperspectral imaging techniques. A commercially available hyperspectral imaging system was modified for the application. We verified that microplastics down to 100 µm could be detected with our modified system.

**Hyperspectral imaging system**

## Default hyperspectral imaging system

**Fig. 1:**
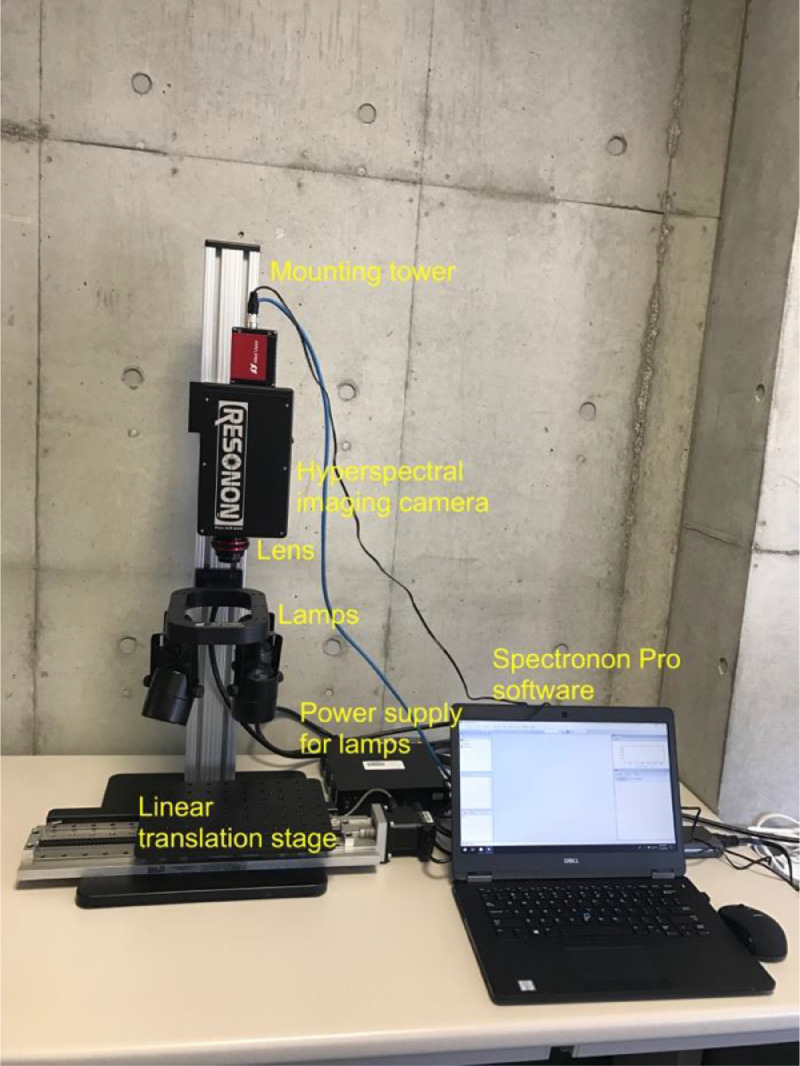
The benchtop default (i.e., before modifications) hyperspectral imaging system used to detect microplastics.

Microplastic detection was based on a benchtop hyperspectral imaging system manufactured by Resonon Inc. (USA; Fig. 1). A hyperspectral camera (Pika NIR-640, Resonon Inc., USA) is mounted on a tower. This camera is incorporated into a two-dimensional InGaAs array detector with a pixel size of 15 µm. A lens with a focal length of 25 mm and a field of view of 21.7° is attached to the camera. Hyperspectral images are collected over NIR wavelengths (900–1700 nm) with a spectral sampling resolution of 2.5 nm. A translation stage on the system is used to transport samples for linear scan. A light assembly (four halogen lamps) that illuminates white light is located on the mounting tower between the camera and the translation stage. A computer is connected to the camera and the translation stage. Image acquisition, control of translation stage movement, and processing of the cube data were conducted using Spectronon Pro software (Resonon Inc., USA).

Hyperspectral data were obtained in reflectance mode because the instrument sensor response and illumination functions were removed. The average dark current noise was first measured while the objective lens was blocked and then subtracted from all data to define zero reflectance. The signal was then scaled by measuring absolute reflectance (reflectance = 1) using a gold-coated polycarbonate filter (GPC0847-BA, Structure Probe, Inc., USA), which is an appropriate substrate for microplastic detection [20]. After adjusting the focus of the objective lens, the near-true aspect ratio was attained through adjustment of the scanning speed and the frame rate (line acquisition rate) considering the linear scan character of the system and conducted with the Spectronon Pro software.

**Fig. 2:**
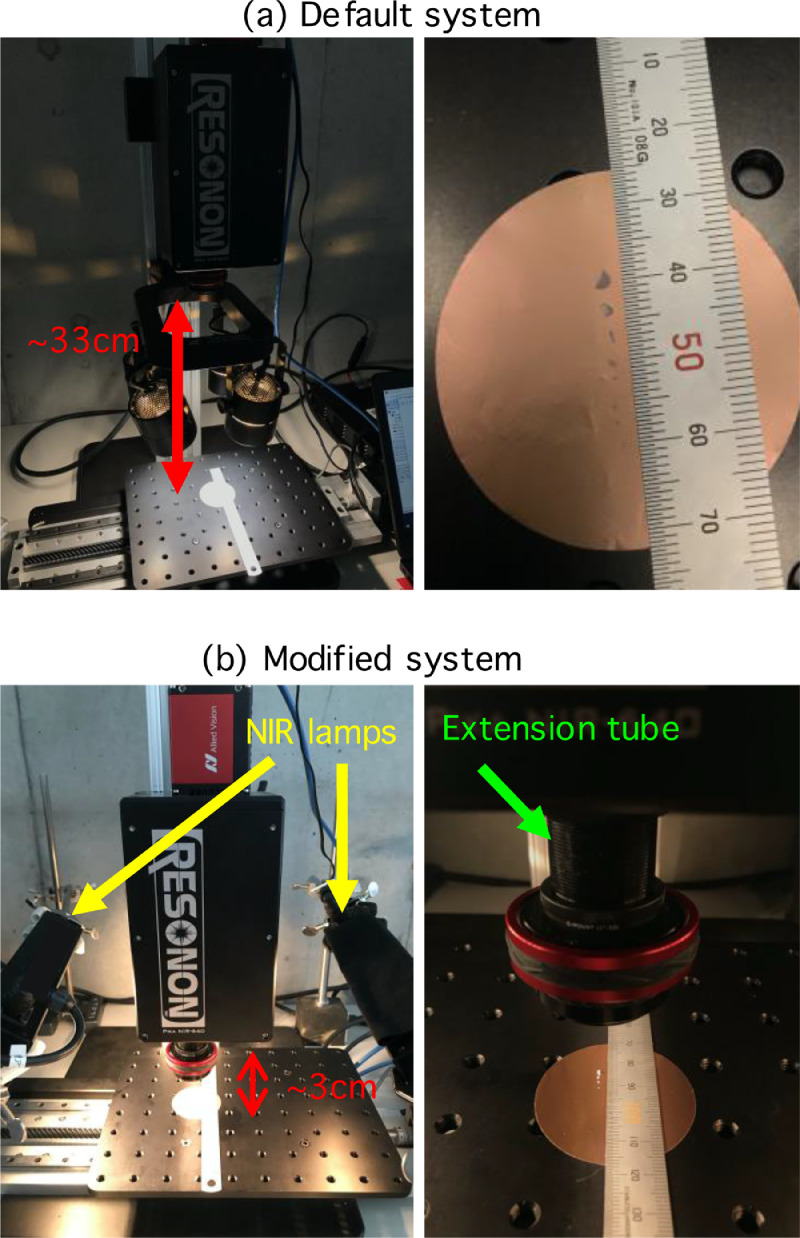
Key working components of the hyperspectral imaging system (a) prior to and (b) after modifications. Polyethylene particles were placed on a gold-coated polycarbonate filter (right panels). A symmetrical set of near-infrared (NIR) lamps was used instead of the default white light lamps and placed on the right side of the camera. An extension tube was attached between the camera and the lens for macro-photography.

The default hyperspectral imaging system was investigated for the minimum detectable size of microplastics. A smooth handling of samples could be assured when the typical camera-to-stage distance was set at ~33 cm (camera-to-lamps = 13 cm, lamps-to-stage = 20 cm; Fig. 2a) because further lowering of the camera and lamps would likely heat the samples, causing physical transformations. Under these conditions, undistorted images could be taken at a scanning speed of 1.5876 cm s^–1^, frame rate of 100 Hz and integration time of 7.814 ms for a scan of 600 lines. With the system in this configuration, the minimal detectable microplastic size was ~250 µm. Thus, to measure smaller microplastics, we modified the hyperspectral imaging system.

## Modifications

The following components of the hyperspectral imaging system were modified: lamp source and position, adjustments to allow for macro-photography, and exposure and aspect ratio.

### Changes to the lamp source and position

**Fig. 3:**
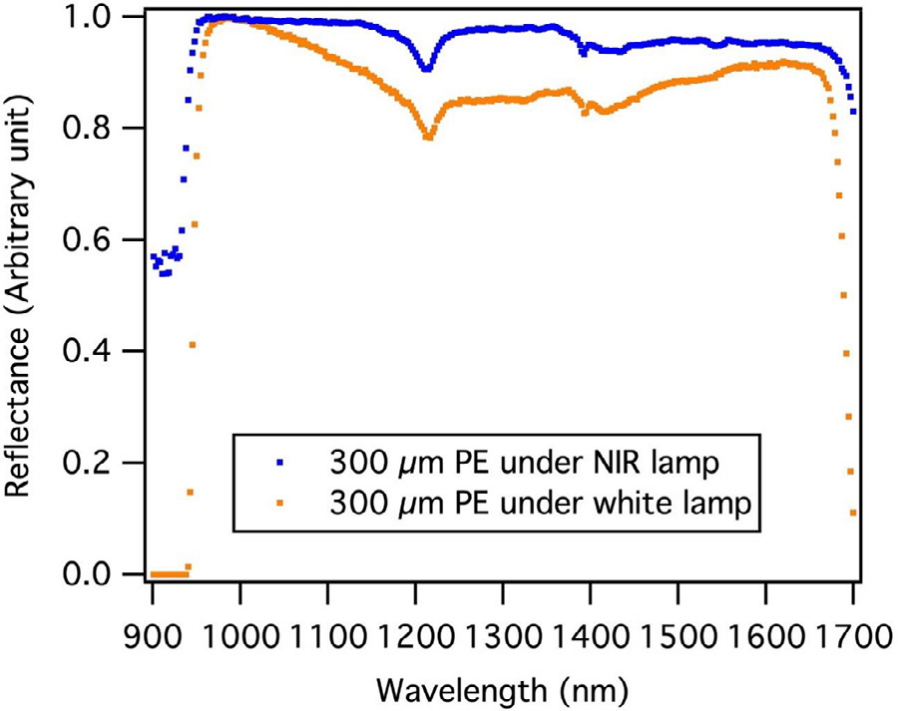
Spectral reflectance of polyethylene (PE) particles measured using the hyperspectral imaging system with a white lamp and a near-infrared (NIR) lamp.

To avoid possible sample heating by the four-lamp assembly, a symmetrical set of two converged-light NIR lamps (LN-200CIR, CCS Inc., Japan) that cover wavelengths of 400–2500 nm with low radiant heat were used. The NIR lamps were placed on the left and right sides of the imaging system to allow the lowering of the camera towards the samples (Fig. 2b). The converged line illumination of the NIR lamps provided enough light intensity while allowing for photography in NIR wavelengths. The spectral responses under different light sources were examined using authentic polyethylene (PE) particles (300 µm) as test samples (Standard Corp., Japan). The PE spectrum showed a more stable reflective baseline of 0.95–0.99 in the 1000–1600 nm wavelength range under the NIR lamps compared to a relatively larger variability of 0.85–0.99 under the four-lamp assembly. As a result, the characteristic absorptive feature at 1540–1550 nm observed under the NIR lamps could not be discerned under the four-lamp assembly (Fig. 3). Moreover, from 1650 nm to 1700 nm, the PE reflectance baseline varied over a relatively small range (0.95 to 0.83) under the NIR lamps, which favors the differentiation of characteristic spectral features. In comparison, the relative reflectance decreased drastically from 0.91 to 0.11 under the four-lamp assembly, leading to a potential loss of spectral features. Our modifications provided better illumination conditions, especially when analyzing small and thin samples with weak characteristic spectral features.

### Adjustment for macro-photography

The system was modified to allow the use of a macro-photography technique for microplastic detection. The distance between the lens and camera was extended by inserting an extension tube (CMV10, Thorlabs Inc., USA; Fig. 2b). This allowed us to decrease the focusing (lens-to-subject) distance and increase the magnification. The camera was then moved down to adjust the focus. Correspondingly, the camera, with lens attached via the extension tube, was placed above the translation stage at a distance of ~3 cm.

### Adjustment of exposure and aspect ratio

During the adjustments for macro-photography, exposure light per pixel decreased. Thus, the amount of light reaching the camera sensor was adjusted by tuning the scanning speed and the frame rate. With the modified system, images with near-true aspect ratio were taken in ~7 s for a scan of 600 lines under a scanning speed of 0.07938 cm s^–1^, frame rate of 100 Hz, and integration time of 7.814 ms. After these modifications, the size of each detection pixel was improved to 14.8 µm.

## Validation of microplastic detection

**Fig. 4:**
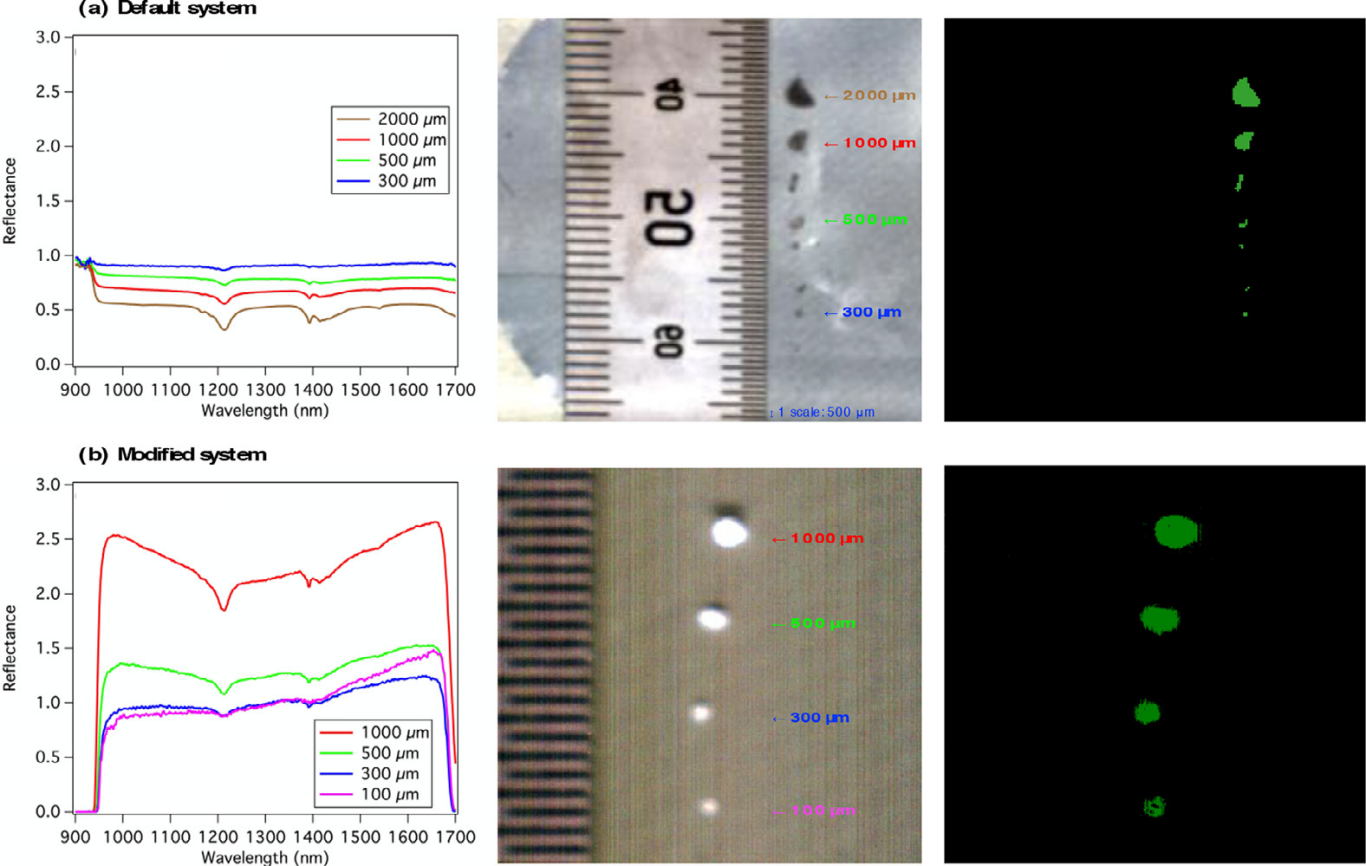
Hyperspectral features of polyethylene particles (2000–100 µm) measured using the (a) default imaging system and (b) modified system. In both (a) and (b): (left) spectra, (middle) RGB images, and (right) classified images based on reference spectra.

The capability of the hyperspectral imaging systems to detect small-size microplastics prior to and after our modifications was validated with authentic PE. Using the default hyperspectral imaging system, PE particles with sizes of 2000 µm, 1000 µm, and 500 µm were detected as 36 (~1500 µm equivalent), 16, and 4 pixels, respectively (Fig. 4a, middle panel). When the particle size was 300 µm, only one pixel was detected and the spectrum showed relatively high noise relative to the baseline; furthermore, the characteristic features at 1150–1250 nm and 1350–1450 nm were dampened (Fig. 4a, left panel). Adopting the Spectral Angle Mapper classification [10], PE particles were identified (Fig. 4a, right panel) using a reference PE spectrum obtained beforehand [20]. To obtain a smooth spectrum (average of 3 pixels or more), we suggest that the default system is only applicable to relatively large (>500 µm) microplastics.

In contrast, PE particles in the size range of 1000–100 µm showed distinct spectral features from the baseline (Fig. 4b, left panel) and unambiguous shapes (Fig. 4b, middle panel) using our modified hyperspectral imaging system. Even at 100 µm, PE spectral features at 1150–1250 nm and 1350–1450 nm were discernable. The classification results showed that PE down to 100 µm could be clearly recognized (Fig. 4c, right panel). The system was verified to be successful to detect 11 common polymer types in the household and industrial sectors and perspectives for quantitative evaluation of microplastic masses [20]. We expect that the modified hyperspectral imaging system could be applied to rapidly detect small microplastics collected on filter substrates. This study provides essential information that moves us toward a better understanding of the spatial and temporal distribution of microplastics in various environments.

### Additional information

Over the past decades, plastics have become some of the most convenient materials for daily and industrial use. However, plastic waste is discharged to the land and ocean, and has led to an emerging pollution issue [3,7]. Microplastics (plastic particles <5 mm in diameter) are produced directly from anthropogenic activities or formed secondarily via physical and chemical processes, such as weathering and decay under ultraviolet light, from primary plastic waste. It has been reported that microplastics adversely affect terrestrial and aquatic ecosystems [2,5]. However, current understanding of microplastic distribution and polymer composition is still limited. One of the major reasons for this is the lack of rapid detection methods.

One of the traditional methods to identify microplastic particles is direct visual observation using optical microscopy [8] where erroneous recognition and miscounting could occur [12]. Other common methods use Fourier transform infrared and Raman spectroscopy [13,18], and these spectroscopic methods are more advantageous than optical microscopy for accurate quantification of both plastic numbers and polymer types [17]. Fries et al. [6] used pyrolysis-gas chromatography coupled to mass spectrometry and scanning electron microscopy to identify plastic polymers and associated additives. Furthermore, Nile Red staining and subsequent detection of fluorescence has been applied to rapidly detect microplastics, but spectroscopic techniques are still needed for polymer type classification [11]. With all of these methods, pretreatments are needed, and these are time-consuming and laborious [16]. Therefore, methods for rapid and convenient quantification of environmental microplastic particles are crucial.

Recently, hyperspectral imaging techniques have been used to detect microplastics. The overtone vibrations of plastic hydrogen–carbon bonds (C–H) show specific spectral reflectance characteristics in near-infrared (NIR) wavelength ranges, showing potential for the quantification of different polymer types [19]. Using a variety of hyperspectral imaging systems with different wavelength ranges, Karlsson et al. [9] reported that plastic particles larger than 300 µm could be detected over wavelengths of 1000–2500 nm. Hyperspectral identification of household and industrial plastics was also verified in the millimeter-size range [1,14]. Recently, it was found that common microplastics around 200 µm could be detected with hyperspectral techniques [15]. However, as the microplastic particles become smaller, the possibility of misidentifying polymer types increases [15]. It is thus necessary to optimize hyperspectral techniques for the rapid and accurate detection of small-sized microplastics.

## Declaration of Competing Interest

The authors declare that they have no known competing financial interests or personal relationships that could have appeared to influence the work reported in this paper.
